# What explains patterns of species richness? The relative importance of climatic‐niche evolution, morphological evolution, and ecological limits in salamanders

**DOI:** 10.1002/ece3.2301

**Published:** 2016-07-26

**Authors:** Kenneth H. Kozak, John J. Wiens

**Affiliations:** ^1^ Bell Museum of Natural History and Department of Fisheries, Wildlife, and Conservation BiologyUniversity of MinnesotaSt. PaulMinnesota55108; ^2^ Department of Ecology and EvolutionUniversity of ArizonaTucsonArizona85721

**Keywords:** Amphibians, climatic‐niche evolution, diversification, ecological limits, morphological evolution, Plethodontidae, rates, salamanders, species richness

## Abstract

A major goal of evolutionary biology and ecology is to understand why species richness varies among clades. Previous studies have suggested that variation in richness among clades might be related to variation in rates of morphological evolution among clades (e.g., body size and shape). Other studies have suggested that richness patterns might be related to variation in rates of climatic‐niche evolution. However, few studies, if any, have tested the relative importance of these variables in explaining patterns of richness among clades. Here, we test their relative importance among major clades of Plethodontidae, the most species‐rich family of salamanders. Earlier studies have suggested that climatic‐niche evolution explains patterns of diversification among plethodontid clades, whereas rates of morphological evolution do not. A subsequent study stated that rates of morphological evolution instead explained patterns of species richness among plethodontid clades (along with “ecological limits” on richness of clades, leading to saturation of clades with species, given limited resources). However, they did not consider climatic‐niche evolution. Using phylogenetic multiple regression, we show that rates of climatic‐niche evolution explain most variation in richness among plethodontid clades, whereas rates of morphological evolution do not. We find little evidence that ecological limits explain patterns of richness among plethodontid clades. We also test whether rates of morphological and climatic‐niche evolution are correlated, and find that they are not. Overall, our results help explain richness patterns in a major amphibian group and provide possibly the first test of the relative importance of climatic niches and morphological evolution in explaining diversity patterns.

## Introduction

Explaining patterns of species richness among clades is a major goal of evolutionary biology and ecology. For example, why do some clades have a single species and others have over a million (e.g., arthropods)? An important approach for answering this question is to test whether particular ecological, morphological, behavioral, or genetic traits are correlated with patterns of diversification (e.g., speciation and extinction over time) and species richness. For example, recent studies have found significant relationships between increased rates of species diversification and occurrence in tropical regions (e.g., Rolland et al. 2014), occurrence on land instead of in water or in marine environments (e.g., Wiens 2015a,b), the presence of defense mutualisms in plants (Weber and Agrawal 2014), and the presence of herbivory in insects (e.g., Wiens et al. 2015). However, individual studies often tend to focus on a single variable, rather than comparing the relative importance of multiple traits on diversification or species richness.

Two traits that might be broadly important in explaining patterns of diversification and species richness are climatic‐niche evolution and the evolution of body shape and size. These traits might be broadly important for several reasons. First, almost all organisms have a climatic distribution (i.e., they occur somewhere, and most places on earth have characteristic large‐scale temperature and precipitation conditions). Similarly, all organisms have morphology (a characteristic shape and size). Second, theory and empirical studies suggest that climatic‐niche divergence may be an important driver of speciation (e.g., Moritz et al. 2000; Kozak and Wiens 2007; Hua and Wiens 2013), as species that occur under divergent climatic regimes may be reproductively isolated from each other if one species cannot tolerate the climatic conditions where the other occurs. Third, morphology may be an important correlate of diversification and patterns of species richness, as different morphologies may be related to the use of different resources (e.g., diet, microhabitat), which might reduce competition and promote coexistence of species, and potentially lead to reproductive isolation and speciation between morphologically divergent populations (e.g., Schluter 2001, 2009; Rundle and Nosil 2005; Nosil 2012), as predicted under the ecological theory of adaptive radiation (e.g., Schluter 2000).

Recent studies have found support both for and against the importance of both variables in diversification. For example, recent studies have supported the importance of climatic‐niche evolution in diversification (e.g., Kozak and Wiens 2010b; Gómez‐Rodríguez et al. 2015; Title and Burns 2015), whereas others have not (e.g., Pyron and Wiens 2013). Similarly, some studies have found strong relationships between rates of body‐size evolution and diversification (e.g., Rabosky et al. 2013) but not others (e.g., Adams et al. 2009). However, few studies (if any) have compared the relative importance of these variables. Thus, even if both niche evolution and morphological evolution were significantly related to diversification or species richness, they might still differ considerably in how much variation in richness or diversification they explained, and under what conditions (note that here and throughout, we mean “explained” in terms of regression analysis, without necessarily implying a direction of causation). Therefore, it is important to perform analyses that directly compare the relative importance of morphological evolution and climatic‐niche evolution in explaining patterns of diversity and diversification among clades in the same group of organisms.

Plethodontid salamanders offer a valuable model system to perform such a comparison. Plethodontidae contains the majority of salamander species (450/682; AmphibiaWeb 2015). Plethodontids have long been an important model system in many research programs in evolutionary biology and related fields (e.g., Bruce et al. 2000). Recent studies have suggested that patterns of variation in net diversification rates among major plethodontid salamander clades are explained by climatic‐niche evolution and not rates of morphological evolution. Adams et al. (2009) found no significant relationship between rates of body‐size evolution and net species diversification rates (estimated following Magallón and Sanderson 2001), nor between rates of body‐shape evolution and diversification. In contrast, Kozak and Wiens (2010b) found a strong relationship between rates of climatic‐niche evolution and rates of species diversification. Together, this pair of results would appear to strongly support the greater importance of climatic‐niche evolution for diversification, relative to morphological evolution.

Rabosky and Adams (2012) reached a very different conclusion, however. They tested for a relationship between species richness (not diversification) and rates of shape and size evolution, using the same data as Adams et al. (2009). They found a significant relationship between both morphological variables and the species richness of plethodontid clades. However, they did not consider rates of climatic‐niche evolution as an explanation for species richness patterns. Rabosky and Adams (2012) also hypothesized that patterns of plethodontid species richness were explained by “ecological limits” on species richness, with patterns of morphological evolution being related to the filling of ecological space and declining rates of species diversification within clades over time. They concluded that these ecological limits made the net diversification rates estimated by Adams et al. (2009) and Kozak and Wiens (2010b) invalid (but see Wiens 2011; Kozak and Wiens 2016). Therefore, they used species richness instead of net diversification rates.

A major conclusion (and assumption) of the study by Rabosky and Adams (2012) is that there are ecological limits on species richness of plethodontid clades that explain the patterns of variation in species diversity among them. However, they did not test any predictions of the ecological limits hypothesis, beyond failing to find a strong, positive relationship between clade age and species richness among clades (a pattern that may have other explanations besides ecological limits; Wiens 2011; Kozak and Wiens 2016). For example, one potential prediction of the ecological limits hypothesis (following from the assumptions of Rabosky and Adams 2012) is that older clades with slower rates of evolution in ecologically important traits will be more saturated with species (i.e., show stronger declines in diversification over time). Therefore, if there are ecological limits on clade richness related to rates of phenotypic evolution, then patterns of decelerating diversification among clades should be strongly associated with interactions between ages of clades and their rates of phenotypic evolution. Alternatively, there might simply be more strongly declining diversification rates in clades with slower rates of morphological evolution, regardless of their age. As far as we know, no previous studies have tested these hypotheses. More generally, despite a large and growing literature on ecological limits, relatively few studies have empirically tested predictions of this hypothesis with relevant ecological data (e.g., Machac et al. 2013; Pinto‐Sánchez et al. 2014; Price et al. 2014). Instead, many studies have inferred ecological limits based on more indirect evidence, such as relationships between clade age and richness (e.g., Rabosky 2009a,b, 2010; Rabosky and Adams 2012; Rabosky et al. 2012), which may be widely misinterpreted (e.g., Kozak and Wiens 2016).

Here, we explore the relationships among species richness and rates of niche and morphological evolution among the major clades of plethodontid salamanders. We also test whether patterns of diversification over time within clades may be associated with ecological limits to clade diversity. We show that patterns of species richness among plethodontid clades are explained primarily by rates of climatic‐niche evolution. Specifically, using phylogenetic multiple‐regression models, we show that morphological variables explain relatively little variation in richness that is not already explained by rates of climatic‐niche evolution. Moreover, we find that rates of morphological evolution are unrelated to rates of climatic‐niche evolution. Finally, we show that apparent slowdowns in diversification within plethodontid clades are unlikely to be explained by saturation or “filling” of morphological or ecological space. Together, these results may offer the first direct comparison of the relative importance of morphological evolution and climatic‐niche evolution in driving patterns of species richness within and among clades, and one of the relatively few tests of the ecological limits hypothesis to directly incorporate ecologically relevant variables.

## Materials and Methods

We tested the hypothesis that species richness of plethodontid clades is significantly and positively related to the rate of climatic‐niche evolution and compared this relationship with that between species richness and rates of size evolution and shape evolution. We then tested whether the size and shape rates still significantly explain species richness in a multiple‐regression model that included the rate of climatic‐niche evolution. We also tested whether these rates of morphological and climatic change are related to each other.

Furthermore, we tested whether declining rates of diversification within plethodontid clades (estimated from the gamma statistic; Pybus and Harvey 2000) are related to an interaction between the ages of clades and their rates of size or shape evolution (as well as rates of climatic‐niche evolution). The latter test was based on the idea that the age of a clade and the rate of trait evolution within a clade can be used as a measure of “niche filling.” For example, if diversification is slowing over time because of niche filling, then older clades should have slower rates of evolution in ecologically important traits and be more saturated with species (i.e., show stronger declines in diversification over time). We also simply tested for a relationship between declining diversification and decreased rates of phenotypic evolution. We note that the gamma statistic used here is the same criterion for slowing diversification used by Rabosky and Adams (2012). In short, even though other tests are possible, we wished to test whether the ecological limits hypothesis was supported given the same evidence available to Rabosky and Adams (2012).

For these analyses, we generally used the same tree and morphological data as Rabosky and Adams (2012), which were generally the same as those used by Adams et al. (2009). We used only the tree with a root age of 61 million years [the only one considered by Rabosky and Adams (2012)]. This tree is based on penalized likelihood (Sanderson 2002, 2003) and assumes a relatively young age for crown‐group plethodontids. However, this tree gives similar ages to those based on multiple nuclear loci analyzed with the Bayesian uncorrelated lognormal approach (Zheng et al. 2011) for major clades within Plethodontidae (for specific comparisons see Fisher‐Reid et al. 2012). The crown age for plethodontids used here is also very similar to that recently estimated by Shen et al. (2016).

For consistency, we also used the same branch length estimates used by Rabosky and Adams (2012), although these differed from those used by Adams et al. (2009). Specifically, for branch lengths of terminal taxa, Adams et al. (2009) used the difference between the crown‐group age and the stem‐group age of each clade. In contrast, Rabosky and Adams (2012) used the stem‐group age for each terminal taxon (but without any justification for this change). The tree and branch lengths used are provided in nexus format in Appendix.

We used the same estimates of species richness and size and shape rates for each of the 15 plethodontid clades as used by Rabosky and Adams (2012), which are originally from Adams et al. (2009). These are provided here in Table 1. In short, Adams et al. (2009) obtained data from museum specimens for 178 species (included in the phylogeny) from seven morphological variables (linear measurements of head, limb, tail, body length, and body width). These data were then analyzed with principal components analysis (PCA). It was found that PC1 represented size, and PCs 2–7 represented shape. Rates of size evolution were therefore based on maximum‐likelihood estimates of *σ* ^2^ for PC1 within each of the 15 clades, whereas rates of shape evolution were based on a single multivariate rate estimate (*σ* ^2^) for PC2–PC7 combined. Estimation of *σ* ^2^ followed O'Meara et al. (2006). Sample sizes of species within each clade are given in Table 1. Note that different clades of plethodontids show considerable variation in size and shape, variation that seems to be well‐captured by these variables and data (e.g., see Fig. 3 of Adams et al. 2009). This variation includes the unusual body shape of *Oedipina*, the variable body shapes among species of the *Pseudoeurycea* clade, the small body sizes of *Nototriton* and *Oedipina*, and the relatively large body sizes of *Aneides*, the *Plethodon glutinosus* group, and the clade of *Gyrinophilus*,* Pseudotriton*, and *Stereochilus*. However, there is also considerable overlap among clades in size and shape. These size and shape variables might be expected to be ecologically important because of potential diet‐related niche partitioning based on head and body size in plethodontid salamanders (e.g., Kryzsik 1979) and because relative head, limb, tail, and body proportions are generally expected to be related to locomotion in different microhabitats (but possibly not in salamanders; see Discussion and Blankers et al. 2012).

**Table 1:** Data on 15 clades of plethodontid salamanders used in this study. Below species richness, we give the number of species sampled for morphology/climate to estimate rates in each clade. Clade ages are in millions of years before present. Units for other variables (rates and gamma) are less straightforward

Clade	Ln‐species richness	Clade age	Climate rate	Size rate	Shape rate	Gamma
Subgenus *Eladinea* (*Bolitoglossa*)	3.828641397 (15/12)	16.3	0.371	0.02225	0.00072	−2.69
Subgenera *Magnadigitata*,* Oaxakia*,* Pachymandra* (*Bolitoglossa*)	3.218875825 (19/20)	19.4	0.163	0.02981	0.00079	−2.65
Subgenera *Bolitoglossa*,* Mayamandra*,* Nanotriton (Bolitoglossa)*	2.833213344 (10/10)	18.8	0.169	0.01832	0.00083	−1.83
*Ixalotriton*,* Lineatriton*,* Parvimolge*,* Pseudoeurycea*	3.931825633 (32/37)	27.6	0.205	0.05166	0.00231	−2.03
*Chiropterotriton*	2.48490665 (7/7)	16.6	0.039	0.0489	0.00043	−2.47
*Oedipina*	3.218875825 (10/13)	18.0	0.178	0.02155	0.00157	−1.74
*Nototriton*	2.564949358 (5/6)	13.5	0.080	0.01509	0.00172	−0.37
*Gyrinophilus*,* Pseudotriton*,* Stereochilus*	1.945910149 (4/4)	23.4	0.024	0.01565	0.00036	−2.17
*Eurycea*	3.583518939 (17/24)	22.7	0.047	0.09287	0.00205	−0.92
Western *Plethodon*	2.197224577 (6/7)	30.5	0.098	0.00423	0.00027	−1.40
*Plethodon cinereus* group	2.302585093 (7/9)	18.1	0.025	0.00615	0.00061	−1.53
*Plethodon wehrlei‐welleri* group	1.945910149 (6/7)	19.9	0.029	0.01385	0.00025	−0.99
*Plethodon glutinosus* group	3.33220451 (18/28)	15.7	0.066	0.01679	0.00062	−2.48
*Aneides*	1.791759469 (5/5)	30.4	0.076	0.01056	0.00027	−1.37
*Desmognathus*,* Phaeognathus*	3.610917913 (17/28)	36.9	0.079	0.03865	0.00072	−1.90

For analyses of climatic‐niche rates, we used the multivariate estimates from Kozak and Wiens (2010b). However, we excluded *Batrachoseps* in the analyses here, as this genus was not included in the analyses of morphological rates by Adams et al. (2009) or Rabosky and Adams (2012). The niche‐rate estimates used here are provided in Table 1. In short, Kozak and Wiens (2010b) estimated rates of climatic‐niche evolution by starting with georeferenced locality data for each of 217 species (included in the phylogeny, and after excluding *Batrachoseps*), and obtaining data for 19 climatic variables for each locality. They obtained the mean value for each variable across the localities for each species. Then, as with the morphological data, they performed PCA and reduced the 19 climatic variables to three PCs that explained 83% of the variation among species. They then estimated *σ* ^2^ for each of these three PCs within each clade and combined all three PCs to obtain a single multivariate estimate of climatic‐niche rate. Sample sizes of species within each clade are given in Table 1.

In summary, very similar methods were employed to estimate the rates of morphological and climatic‐niche evolution. Importantly, the same time‐calibrated phylogeny and methods (*σ* ^2^ of PCs) were used to estimate rates for each type of variable in each clade. Furthermore, the sample sizes for each variable type within each clade were not always identical, but were strongly associated (*r* ^2^ = 0.89; *P *<* *0.0001). Similarly, sample sizes within each clade were strongly related to the total number of species within each clade (morphology: *r* ^2^ = 0.79; *P *<* *0.0001; climate: *r* ^2^ = 0.68; *P *<* *0.0001). We also note that Kozak and Wiens (2010b) showed that estimated rates of evolution do not seem to be influenced by incomplete sampling within each clade. Finally, as the same plethodontid topology is used to estimate rates for each dataset, the species limits assumed in each analysis are also the same.

Regression analyses of species richness and rates among clades were performed using the phylogenetic generalized least‐squares approach (PGLS; Martins and Hansen 1997), as implemented in the R package *caper*, version 0.5.2 (Orme et al. 2013). For *caper* analyses, the maximum‐likelihood value of lambda was estimated, and kappa and delta were both fixed at 1. We estimated regression models with ln‐transformed richness as the dependent variable and various combinations of independent variables, including (1) climatic‐niche rates alone; (2) size rate alone; (3) shape rate alone; (4) size and shape rates together; (5) size rate and climatic‐niche rate; (6) shape rate and climatic‐niche rate; and (7) size, shape, and climatic‐niche rate. We calculated the AIC of each model, and evaluated whether models including one or both morphological rates had substantially better fit than models including only climatic‐niche rate. An AIC difference of four or greater between competing models was considered to show substantially better fit of one over another (Burnham and Anderson 2002). We also tested whether rates of size and shape evolution are significantly related to rates of climatic‐niche evolution.

Finally, we used PGLS to test whether declining diversification rates within clades over time were related to an interaction between rates of morphological evolution and the ages of clades. We used estimates of gamma from Rabosky and Adams (2012) as the dependent variable and then included as independent variables either (1) age, rate of shape evolution, and the interaction of these two variables; or (2) age, rate of size evolution, and their interaction. We then repeated these analyses after replacing rates of morphological evolution with the rate of climatic‐niche evolution. We also performed a simpler version of this analysis, and merely tested whether slower rates of size and shape evolution were associated with a stronger slowdown in diversification, as indicated by more negative values of gamma. Values of gamma and crown‐group age for each clade are provided in Table 1.

## Results

**Figure 1:**
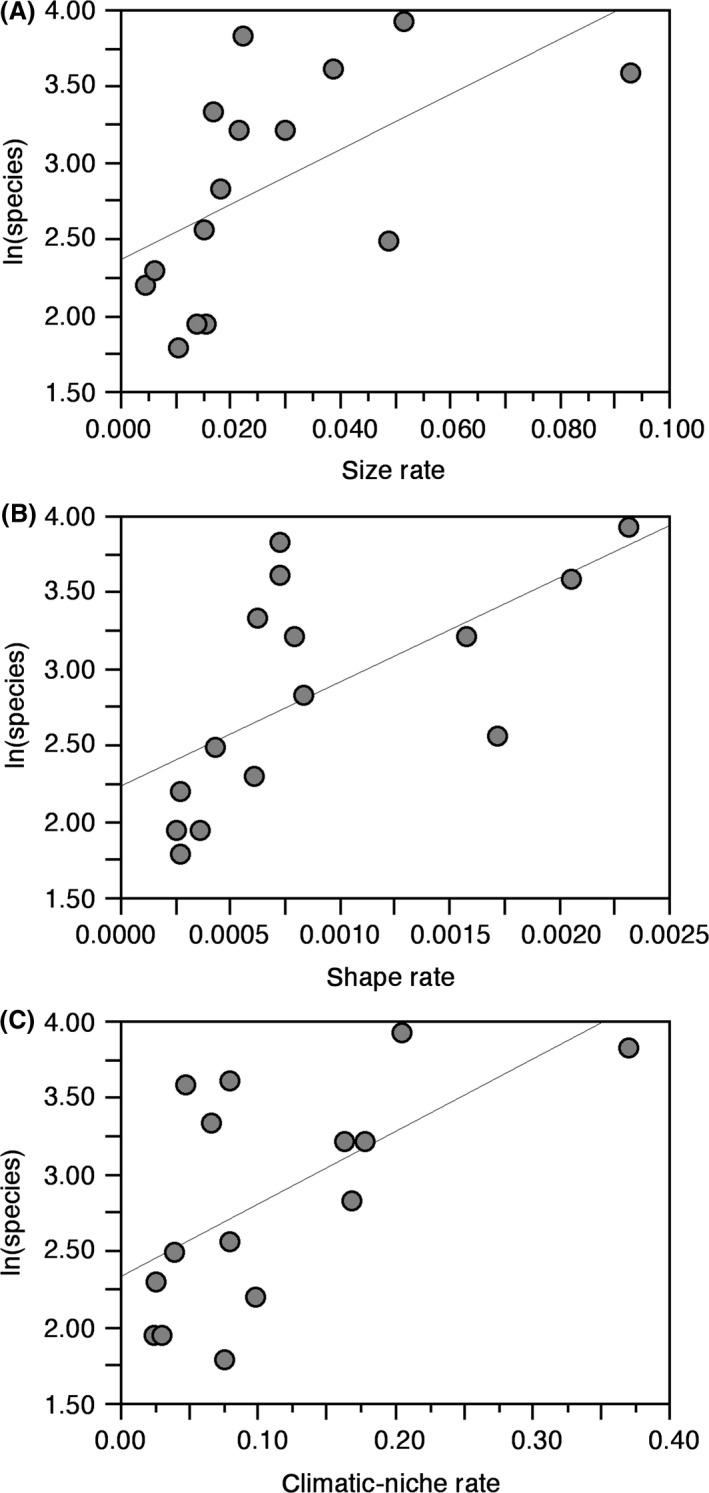
Plots of species richness and evolutionary rates for plethodontid salamanders (data in Table 1), including (A) size rate, (B) shape rate, and (C) climatic‐niche rate. The thin line is the ordinary least‐squares regression line (drawn for illustration only), but the results are based on phylogenetic generalized least‐squares analysis (Table 2).

**Figure 2:**
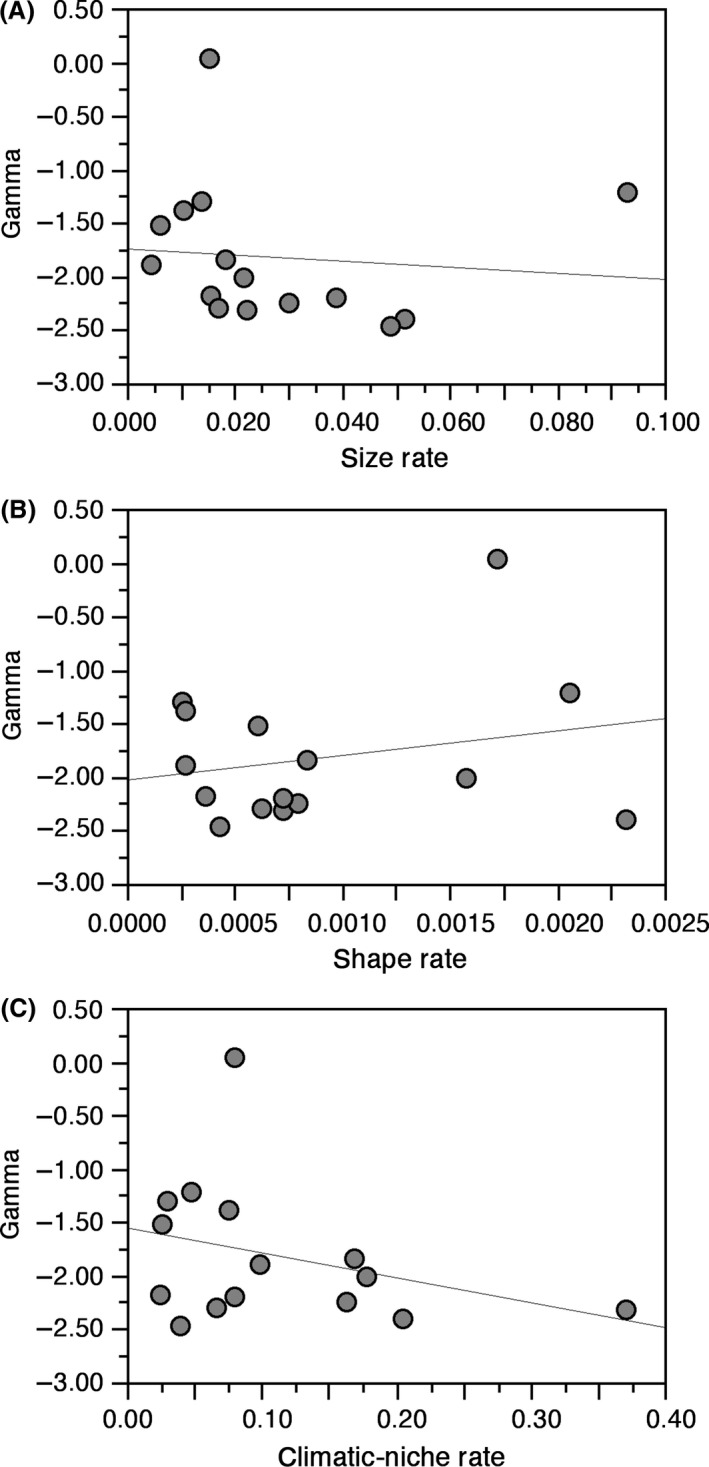
Plots of declining diversification rates within clades (gamma) and evolutionary rates for plethodontid salamanders (data in Table 1), including (A) size rate, (B) shape rate, and (C) climatic‐niche rate. The thin line is the ordinary least‐squares regression line (drawn for illustration only), but the results are based on phylogenetic generalized least‐squares analysis (Table 2).

The results of the PGLS analyses (Table 2) show that rates of climatic‐niche evolution have a much stronger relationship with patterns of species richness than rates of size or shape evolution (Fig. 1). Rates of climatic‐niche evolution explain almost two‐thirds of the variation in species richness among plethodontid clades (*r* ^2^ = 0.58), whereas size and shape rate each explain closer to one‐third (*r* ^2^ = 0.30 and 0.38, respectively). Most importantly, even though a model including all three variables had the best fit (AIC = 15.04822), this model was not substantially better than one based on climatic‐niche rate alone (AIC = 18.26653; ΔAIC < 4.0). There is no significant relationship between size or shape rate and rates of climatic‐niche evolution (Table 2). Furthermore, despite considerable variation in the ages of clades and in rates of phenotypic evolution among them (Table 1), we found no evidence that declines in diversification rates over time within clades are associated with measures of niche filling. Specifically, values of gamma show no association with the interaction between age and rates of morphological evolution (Table 2). Similarly, values of gamma are unrelated to an interaction between age and the rate of climatic‐niche evolution (Table 2). Finally, we found no significant relationship between values of gamma and rates of size, shape, or climatic‐niche evolution (Table 2). Although the relationship between gamma and size rate approaches significance, more negative values of gamma (i.e., more strongly slowing diversification) are associated with faster rates of size evolution (Fig. 2). This is the opposite of what would be expected if slowing rates of diversification were associated with slowing rates of phenotypic evolution, as predicted under the ecological limits hypothesis.

**Table 2:** Results of PGLS analyses of 15 plethodontid clades. Climate rate, size rate, and shape rate refer to estimated rates of evolution in these traits within clades

Variables	r^2^	P	AIC
Ln(species) ~ size rate	0.2994	0.0456	24.3910
Ln(species) ~ shape rate	0.3850	0.0173	22.8275
Ln(species) ~ climate rate	0.5795	0.0013	18.2665
Ln(species) ~ shape rate + size rate	0.4666	0.0478	23.1199
Ln(species) ~ climate rate + shape rate + size rate	0.7696	0.0048	15.0482
Ln(species) ~ climate rate + shape rate	0.7188	0.0020	15.4384
Ln(species) ~ climate rate + size rate	0.7113	0.0022	15.7513
Size rate ~ climate rate	0.0073	0.9294	99.9315
Shape rate ~ climate rate	0.1275	0.2774	25.2822
gamma ~ age × shape rate + age + shape rate	0.4166	0.2033	26.4472
gamma ~ age × size rate + age + size rate	0.3230	0.3566	28.2318
gamma ~ age × climate rate + age + climate rate	0.2475	0.5183	29.5007
gamma ~ size rate	0.2628	0.0678	25.2547
gamma ~ shape rate	0.0103	0.9020	28.7885
gamma ~ climate rate	0.2268	0.0995	25.8266

## Discussion

In this study, we test whether patterns of species richness in the largest clade of salamanders are explained primarily by rates of divergence in climatic niches or morphology. Additionally, we test whether there is evidence that declining diversification rates are associated with slowing rates of size or shape evolution, as predicted under the ecological limits hypothesis. Our results show that most variation in species richness in plethodontid salamanders is explained by rates of climatic‐niche evolution, not rates of shape or size evolution. Further, we show that there is little evidence for declining diversification rates associated with declining rates of shape or size evolution, and little evidence for ecological limits on plethodontid species richness overall. In the sections below, we discuss these two conclusions in turn.

### Climatic‐niche evolution versus morphological evolution

In this study, we provide possibly the first test of whether patterns of species richness are more strongly related to morphological evolution or to climatic‐niche evolution. Our results strongly support rates of climatic‐niche evolution as more important. Indeed, rates of climatic‐niche evolution explain >50% of the variation in richness among plethodontid clades. We acknowledge that these results might not apply to other groups. For example, plethodontids may be unusual in that their morphology (i.e., size and shape) is somewhat uncoupled from their ecology (i.e., microhabitat; Blankers et al. 2012). This uncoupling might reduce the potential linkages between morphology and diversification that may be more common in other groups. Nevertheless, an alternate possibility is that climatic‐niche divergence is generally more important in diversification and species richness. This might be because climatic‐niche divergence is an important driver of speciation, by preventing gene flow between populations in different climatic regimes (e.g., Mortiz et al. 2000; Kozak and Wiens 2007; Hua and Wiens 2013). In contrast, divergence in size and shape may be very important ecologically (e.g., in reducing competition and facilitating species coexistence in sympatry; Schluter 2000), but they might play less of a direct role in most speciation events within a given group, with geographic separation being more frequently important instead. Indeed, allopatric speciation seems to be important in animals in general (e.g., Coyne and Orr 2004) and in plethodontids in particular (e.g., Kozak and Wiens 2006). Again, similar studies in other organisms are needed to address the generality of these patterns.

We acknowledge that our results may be unsurprising given the previous studies showing that net diversification rates were related to climatic‐niche evolution and not rates of size and shape evolution (Adams et al. 2009; Kozak and Wiens 2010b). Rabosky and Adams (2012) cited the study by Kozak and Wiens (2010b), but did not consider climatic niches. Furthermore, Rabosky and Adams (2012) concluded that diversification rates and richness are tightly linked (their Fig. 2), suggesting that climatic‐niche evolution should be related to species richness, not just diversification rates.

We note we have not inferred the direction of causation between richness and climatic‐niche evolution (we merely use “explain” statistically). However, even though clades with greater species richness should have more climatic‐niche divergence among species based on sampling alone, this would not explain higher rates of change per unit time, as found here. Furthermore, analyses of rates of niche divergence and rates of net species diversification also support our inference that the number of species alone does not explain climatic‐niche divergence (Kozak and Wiens 2010b).

### Ecological limits on clade richness

Many studies have argued that there are ecological limits on clade richness, such that species richness fails to increase in clades over time, supposedly due to competition for limited resources. However, many of these studies include little or no ecological data (e.g., Rabosky 2009a, 2010; Rabosky et al. 2012). Moreover, they do not address whether patterns of decelerating diversification within clades over time are actually associated with the “filling” of ecological space. Rabosky and Adams (2012) suggested that there were ecological limits on species richness in plethodontid clades related to rates of morphological evolution. Several lines of evidence counter this hypothesis.

First, our results here show no evidence that slowing diversification within clades is related to an interaction between clade age and rates of shape or size evolution (Table 2), demonstrating that older clades undergoing slower rates of morphological and climatic‐niche evolution are not more “saturated” with species than younger clades having faster rates of change in these traits. Moreover, there is no strong relationship between gamma and rates of size and shape evolution (Table 2), showing that clades with slower rates of morphological and climatic‐niche evolution are not more saturated with species than clades with faster rates of phenotypic change. Although the relationship between size rate and gamma approaches significance, size rates are actually faster in clades with more negative gamma (Fig. 2). This is the opposite of the pattern expected if size rates have slowed in clades with declining diversification rates.

Second, the decoupling of ecological radiation from size and shape evolution in plethodontids is also problematic for the ecological limits hypothesis. For example, Rabosky and Adams (2012) stated that, “If there are strong diversity‐dependent controls on species richness within clades, then clades with higher phenotypic rates may occupy increasingly broad regions of ecological space.” Yet, given that divergence in size and shape is largely unrelated to microhabitat usage in plethodontids (Blankers et al. 2012), greater phenotypic divergence in these traits may not be strongly related to broader ecological space. Note that the analysis of Blankers et al. (2012) did not include all morphological variables that might be relevant to their ecology, but did include the same morphological variables used by Rabosky and Adams (2012) to estimate phenotypic rates.

Third, the results of Rabosky and Adams (2012) actually show that phenotypic disparity increases within clades over time (their Fig. 1C). This result alone strongly suggests that morphological “space” within these clades has not become saturated over time. Therefore, this result contradicts their supposition that there are ecological limits on clade richness related to these traits.

Fourth, phylogenetic analyses of the three major clades of plethodontids that occur sympatrically in eastern North America (*Desmognathus*,* Plethodon*, Spelerpinae; Kozak et al. 2009) show patterns that strongly contradict the predictions of ecological limits on richness related to morphological evolution. These analyses showed that these clades have undergone parallel and convergent patterns of morphological evolution, such that sympatric species in different clades are actually more morphologically similar than expected by chance. Thus, morphological similarity of species does not preempt the diversification of coexisting clades.

Finally, studies have repeatedly shown evidence that both local and regional species richness increase over time in plethodontids (e.g., Wiens et al. 2007; Kozak and Wiens 2010a, 2012). These include studies of elevational richness in Middle America (Wiens et al. 2007) and eastern North America (Kozak and Wiens 2010a) and studies of both local and regional richness across the geographic range of Plethodontidae (Kozak and Wiens 2012). The observation that species richness seems to increase over time contrasts with the idea that these communities, habitats, and regions have become saturated with plethodontid species over time, as predicted by the ecological limits hypothesis. Importantly, we have also shown that sympatry between major plethodontid clades seemingly decreases their rates of net diversification (Kozak and Wiens 2010b), as expected if competition for limited ecological resources slows diversification within clades. Yet, the observation that diversification seemingly slows over time is not evidence that it stops completely or that ecological limits explain variation in species richness among clades and regions, especially as richness apparently increases over time at both local and regional scales in plethodontids (for a similar example in hylid frogs, see Wiens et al. 2011). Indeed, patterns of declining diversification within clades were previously shown to be uncoupled from richness patterns among plethodontid clades (Kozak and Wiens 2010b).

In summary, we find little evidence that species richness in plethodontids is strongly limited by “carrying capacity” or “ecological limits” related to these morphological variables. Instead, many lines of evidence contradict this idea. These results also call into question the idea that analyzing net diversification rates was inappropriate in plethodontids in the first place (and it is not clear that ecological limits make estimates of *net* diversification rates invalid, even if they caused *instantaneous* diversification rates to change over time; Wiens 2011). Indeed, Rabosky et al. (2013) abandoned the approach used by Rabosky and Adams (2012) and analyzed net diversification rates instead of species richness among clades (but this time supporting a relationship between rates of diversification and morphological evolution). This was the same approach used by Adams et al. (2009) to reject a relationship between morphological rates and diversification. More broadly, these results also suggest that the general approach used by Rabosky and Adams (2012) to argue for ecological limits in plethodontids (i.e., the absence of a positive relationship between clade age and richness) could be misleading, at least without support from more detailed ecological analyses within and among clades. This has implications for many studies using the age‐richness relationship among clades as the primary test for ecological limits (e.g., Rabosky 2009a,b, 2010; Rabosky et al. 2012). Recent simulations also show that this approach may be highly problematic (Kozak and Wiens 2016).

## Conclusions

Finding the ecological, morphological, and genetic correlates of large‐scale patterns of diversification and species richness is an important goal of evolutionary biology. Our results from a major clade of amphibians show that climatic‐niche evolution may be more important than morphological evolution in explaining these patterns. Whether this is a general pattern (or an unusual one in plethodontids) remains to be seen. More broadly, our results illustrate the potential shortcomings of considering only a single predictor variable when trying to understand patterns of richness and diversification. Similarly, our results here contribute to many other lines of evidence arguing against ecological limits on species richness in plethodontids related to morphological evolution. These latter results underscore the need for objective, detailed ecological analyses to test hypotheses of ecological limits, rather than merely testing relationships between clade age and richness.

## Conflict of Interest

None declared.

## Phylogeny and branch lengths used, in nexus format. Full names of each taxon are given in Table 1 and are listed in the same order as the taxa in the tree below.

((((((Eladinea:23.490234,(Magnadigitata:21.850650,Bolitoglossa:21.850652):1.639585):6.825100,Ixalotriton:30.315336):5.305069,Chiropterotriton:35.620404):3.654186,(Oedipina:30.930918,Nototriton:30.930918):8.343672):10.371888,(Gyrinophilus:37.570266,Eurycea:37.570266):12.076196):11.353538,((Western_Plethodon:41.903526,(cinereus_group:27.089525,(wehrlei_group:23.154290,glutinosus_group:23.154290):3.935235):14.814001):6.233287,(Aneides:41.840332,Desmognathus:41.840333):6.296479):12.863188):0.
